# Supplementary material for: Complexome profiling of the Chlamydomonas psb28 mutant reveals TEF5 as an early PSII assembly factor
Source: Plant Cell. 2025 Mar 17;37(6):koaf055. doi: 10.1093/plcell/koaf055 (PMC12164590; doi:10.1093/plcell/koaf055)
Supplement: koaf055_Supplementary_Data [file koaf055_supplementary_data.zip › psb28_interactive.zip!/plots/intensity/qualityPlots/peptides/NUOP1_Cre13.g571150.t1.2.html]

### Cre13.g571150.t1.2

Trivial name: NUOP1  
SeqCoverage [median]: 55.80  
Mass: 9.98 kDa  
  
 

| Sequence | MissedCleavage | Mass [Da] | wtIntensity | muIntensity | Score |
| --- | --- | --- | --- | --- | --- |
| AAADWEDEALKR | 1 | 1373.66 | 10280.40 | 14477.83 | 82.36 |
| ELFAANQYK | 0 | 1082.54 | 4581.52 | 4255.41 | 158.12 |
| GYAIPEDRK | 1 | 1047.53 | 374.22 | 221.23 | 45.16 |
| GYAIPEDRKELFAANQYK | 2 | 2112.06 | 659.50 | 3489.47 | 43.30 |
| KELFAANQYK | 1 | 1210.63 | 7390.79 | 520.14 | 130.75 |
| KGYAIPEDR | 1 | 1047.53 | 1034.16 | 353.40 | 76.83 |
| KGYAIPEDRK | 2 | 1175.63 | 1489.57 | 1670.42 | 85.96 |
| KGYAIPEDRKELFAANQYK | 3 | 2240.16 | 1346.30 | 0.00 | 41.87 |
| RPYIHGAGMIAGYYLFK | 1 | 1956.01 | 982.14 | 0.00 | 40.80 |
|  |  |  | 28138.6 | 24987.9 |  |
